# A Preliminary Investigation of Research Collaboration Through Scientific Paper Co-authorship in the Gulf of Mexico

**DOI:** 10.1007/s12237-025-01616-y

**Published:** 2025-10-04

**Authors:** Juliet Vallejo, Evelyn Roozee, Dongkyu Kim, Andrew M. Song, Christopher A. Gabler, Jasper de Vries, Antonia Sohns, Gordon M. Hickey, Owen Temby

**Affiliations:** 1 School of Earth, Environmental, and Marine Sciences, The University of Texas Rio Grande Valley, Brownsville, TX USA; 2 Department of Natural Resource Sciences, McGill University, Sainte-Anne-de-Bellevue, QC Canada; 3 School of Political Science, Public Affairs, Legal & Security Studies, The University of Texas Rio Grande Valley, Edinburg, TX USA; 4 Faculty of Arts and Social Sciences, University of Technology Sydney, Ultimo, NSW Australia; 5 Strategic Communication Group, Wageningen University, Wageningen, Netherlands

**Keywords:** Research networks, Gulf of Mexico, Science policy, Co-authorship, Collaboration

## Abstract

It is well known that publications with collaborators from external institutions increase citations. This effect scales with spatial distance. There are also many barriers to long-distance collaborations, including linguistic differences, funding constraints, and the incremental costs of remote collaboration. This paper uses the Gulf of Mexico as a case study to examine long-distance research collaboration because it consists of three countries with diverse development levels and two prominent diplomatic languages, within a singular regional ecosystem of tremendous natural and economic value. This paper uses bibliometric network analysis to examine scientific research article co-authorship in the Gulf of Mexico from 2000 to 2018. The results reveal that, although inter-organizational co-authorship has increased, significant fragmentation exists between the U.S.A, Mexico, and Cuba. Large differences in technological and organizational proximity as well as research capacity between US and Mexican states in the Gulf of Mexico may make collaboration more difficult compared to other transboundary settings, such as the US-Canadian border. Centrally located organizations in the network, such as NOAA, have played a prominent role in cross-institutional research, suggesting a capacity to bridge political entities in the Gulf of Mexico.

## Introduction

Studies of scientific co-authorship patterns show that publishing with collaborators from external institutions increases readership and citations. This effect scales: spatially distant co-authorships are cited more often than closer co-authorships (Glanzel & De Lange, 2002; Nomaler et al., 2013; Abramo et al., 2020). International co-authorships are cited more than those occurring within national political borders (Katz & Hicks, 1997; Nomaler et al., 2013). Reasons offered for this vary, with Wagner et al. (2019) showing an “audience effect,” where international co-authorships result in a larger citing community of scholars. The benefits of international co-authorship are more expansive than publication reach. The collaborative process enables scholars in different countries to share regionally located expertise and resources, as well as gain access to distant study sites (Katz & Martin, 1997; Kim, 2006; Rolfe et al., 2004). This last benefit is especially relevant for the study of coastal ecosystems that span political boundaries, since this can include university systems and national budgets with differing capacities to support ecosystem science (King, 2004).

Governments have identified long-distance inter-institutional collaboration as a priority through significant funding opportunities. He (2009) highlights the efforts of the National Natural Science Foundation of China to support collaborative agreements with science foundations and research institutes in dozens of countries. Hoekman et al. (2010) describe the European Union’s initiative for a European Research Area pooling resources and improving scientific collaboration among institutions in EU member states. In the US coastal context, the National Oceanic and Atmospheric Administration (NOAA) has created multi-university institutes known as cooperative institutes (NAO 216-107A). The federal government provided hundreds of millions of dollars to facilitate research between cooperative institutes and NOAA offices. Three of these cooperative institutes are located in, and principally research, the Gulf of Mexico: the Center for Coastal and Marine Ecosystems, the Cooperative Institute for Marine and Atmospheric Sciences, and the Northern Gulf Institute. The development of government funding and policy supporting collaborative research plays a key role in the increasing trend of co-authorships. Although co-authorship trends vary across fields, generally, the number of co-authorships (including international co-authorships) has steadily increased over the last three decades (Elango & Rajendran, 2012; Gorraiz & Schloegl, 2008; He, 2009; Lancho-Barrates & Cantu-Ortiz, 2019; Song et al., 2015). Song et al. (2015) demonstrated this phenomenon in the US Great Lakes. However, no research has examined co-authorship trends in the Gulf of Mexico despite the region’s rich and economically valuable ecosystem.

The Gulf of Mexico is a deep, semi-enclosed oceanic coastal basin with shores bordering the U.S.A., Mexico, and Cuba. The Gulf region is one of the world’s most productive ecosystems, featuring a rich diversity of habitats and species, and it provides vital services such as oil and gas production, tourism, fisheries, and maritime transport (Cruz & McLaughlin, 2008). Gulf of Mexico resources are essential to its regional economies and are heavily impacted by human pressures, such as extensive shoreline development, water engineering systems, and offshore oil development (Alvarez Torrez et al., 2017). Additionally, the region is vulnerable to changing weather systems and environmental hazards, such as hurricanes and the second-largest dead zone in the world due to nutrient loading from the Mississippi River (Alvarez Torrez et al., 2017). These hazards significantly impact economic security, human health, safety, and the region’s habitats (Wondolleck & Yaffee, 2017). Simultaneously, the environmental policy and management capacity of the nations surrounding the Gulf of Mexico vary substantially (Healy et al., 2014).

 In the Gulf of Mexico, due to discrepancies in scientific capacity and complex political relations, collaborative research between Cuba, Mexico, and the U.S.A. has reportedly been difficult to achieve (Cruz & McLaughlin, 2008). Hoekman et al. (2010) found that physical distance and linguistic differences negatively affect co-authorship research activities. Katz and Martin (1997) highlight the challenges of project administration at institutions with different management cultures. Another persistent challenge has been the reluctance of governments to fund research at institutions in different countries (Banchoff, 2002), even if the scale of the research crosses national borders.

This paper presents a preliminary view of inter-institutional and international co-authorship research with an interconnected perspective on the Gulf of Mexico ecosystem. It uses bibliometric analysis examining which organizations co-produce scientific research that supports Gulf of Mexico environmental governance and the extent to which organizations collaborate across organizational, institutional, and jurisdictional boundaries. This initial assessment offers insight into co-authorship trends within the Gulf of Mexico region, highlighting central collaborative organizations and underdeveloped connections.

## Methods

### Bibliometric Analysis

Bibliometric analysis is a quantitative method that summarizes the main characteristics of related literature (Zhang & Liang, 2020). It can be used to understand historical trends of a research field and identify gaps that should be addressed in future research. A bibliometric analysis of co-authorship is a practical, verifiable, and unobtrusive method for approximating collaborations in scientific research (Glänzel & Schubert, 2004; Katz & Martin, 1997; Klenk et al., 2010a, 2010b; Melin & Persson, 1996). We acknowledge that bibliometric analysis is only a partial indicator of collaborative activity focused on the final product of one specific type of formal collaboration. However, the methodology of this study follows the established contours of assumption, procedures, and caveats previously used in similar types of analyses of transboundary regions (see Song et al., 2015).

### Data Collection

**Fig. 1:**
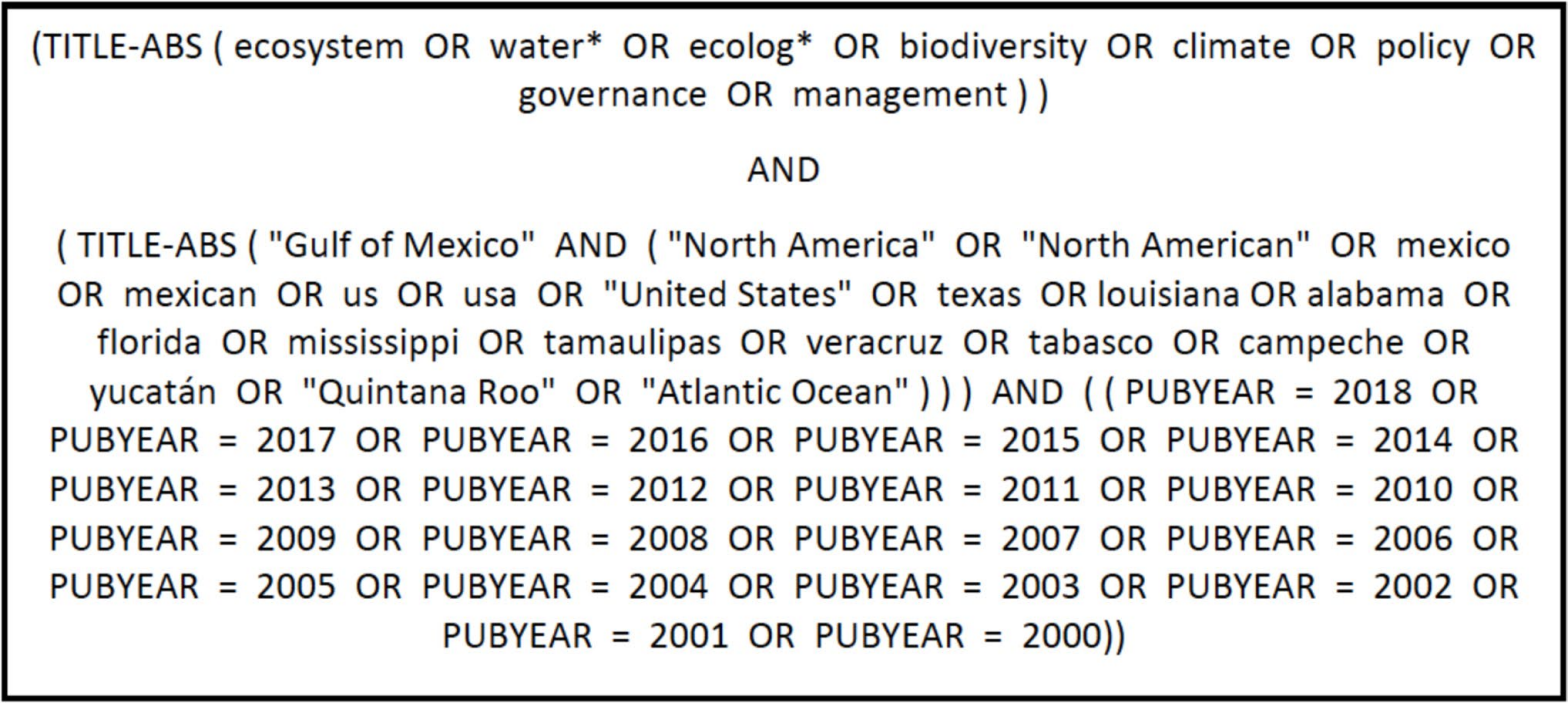
Search terms used in the SCOPUS database

Published papers with inter-organizational co-authors were used as a proxy for scientific collaboration. Data were obtained from Elsevier SCOPUS, a reputable bibliographic database known as one of the most comprehensive sources for peer-reviewed journals across all fields, with over 20,000 journals worldwide (Gorraiz & Schloegl, 2008). The search criteria comprised two parts: search theme and geographic location. The *thematic* search focused on Gulf of Mexico science with an interconnected perspective of the regional ecosystem (e.g., search terms such as *ecosystem*, *watershed/way*, *climate*, *policy*, and *management*). This was combined with *geographic* search criteria (e.g., search terms such as *Gulf of Mexico*, *North America*, *Mexico*, *Cuba*), resulting in papers that met both criteria in either the title or abstract (see search terms in Fig. 1). The search focused on 18 years from 2000 to 2018 and included articles, notes, reviews, and editorials. Our search produced a total of 8001 publications that met the selection criteria, and an initial manual quality control was applied to check for relevance and duplication. A set of 4606 published articles was kept for further analysis.

The dataset contains basic information about each publication, including journal title, publication year, article title, author name(s), and author affiliation address(es). To identify inter-organizational collaborative scholarship, the original SCOPUS data was processed largely through three steps. First, we coded the dataset using Stata string functions to identify the geographic location of an author and affiliated organizations. Second, we manually coded the remaining data points that Stata string functions failed to code. All Mexican affiliation information was translated and coded by the Center for Survey Research and Policy Analysis at the University of Texas Rio Grande Valley. Third, further quality control followed in removing authorship pairings with co-authors from identical organizations and removing single authorships from analysis. Starting with 15,383 authors, removing duplication or single authors reduced the authorship list to 13,064.

Once these steps were completed, our data set included a total of 4245 collaborative research articles, with 2490 authoring organizations in 82 countries. The 2490 organizations were measured for the number of collaboration links to other organizations (i.e., the number of other organizations that authors of those organizations collaborated with) and the total links to other organizations, including redundant links. The organizational affiliations of the authors were categorized by geographical location and by institution type to create two sets of network files. In the first set, the node file categorizes the data by geographical location, and the edge file depicts the inter-organizational co-authorships between them. The second set categorizes the data by institution type and the inter-organizational co-authorships between them. The identified geographic locations were as follows: US national government scale, Mexican national government scale, Cuban national government scale, entities located within Cuba, US and Mexican states bordering the Gulf of Mexico, US states outside of the Gulf of Mexico region, Mexican states outside of the Gulf of Mexico region, entities outside the Gulf of Mexico, and inter-governmental entities (e.g., United Nations Environment Program). For institution types, we coded 21 categories, including inter-governmental organizations, a US-based set of federal agencies and sub-agency regional bureaus, sub-national agencies (e.g., state or municipality agencies), educational institutions, non-government/not-for-profit organizations, and private organizations, and the equivalent set for Mexico, Cuba, and countries outside of the Gulf of Mexico. A full list of geographic locations and institutional affiliations is provided in the publicly available dataset archived in ScholarWorks@UTRGV (Temby et al., 2025). The two sets of edge and node files were used in the VOSviewer program to create two density maps that represent the inter-organizational knowledge production network, one organized by political jurisdiction and one by institution type.

## Results

### Publication Output, Journals, Inter-organizational Collaboration

**Fig. 2:**
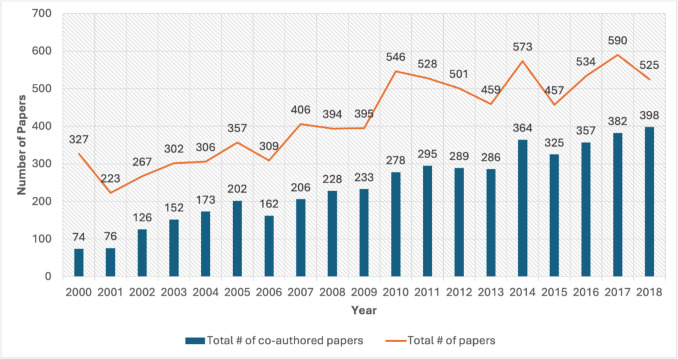
The total number of Gulf of Mexico–related research papers and the number of inter-organizationally authored papers produced by year from 2000 to 2018. Data source: Scopus (Elsevier), 2000–2018; curated as in Temby et al. (2025) [dataset]

Collaborative research papers on the Gulf of Mexico published between 2000 and 2018 were distributed among 1059 journals. Table 1 lists the top 15 journals by the number of collaborative publications, with *Proceedings of the Annual Offshore Technology Conference* being the most prominent venue with 123 papers (2.9% of the total 4,245 published collaborative papers). Over time, there is an increasing trend for both the total number of Gulf of Mexico–related research papers produced and the number of inter-organizationally co-authored papers (Fig. 2). NOAA was the most common organizational affiliation, with participating authorship on 683 published papers (5.2% of all authorship on co-authored publications), connected to 431 different organizations with a total strength of 1325 links (Table 2). The top seven organizations each published 200 or more collaborative papers. These seven organizations consisted of two US national government agencies, four US universities, and one from Mexico. Among the nine American universities listed in Table 2, six have research funded through NOAA cooperative institutes (CIs).

**Table 1:** Top 15 journals publishing collaborative research in the Gulf of Mexico region by number of papers (2000–2018). The reported percentage is out of the total 4245 published collaborative papers. Data source: Scopus (Elsevier), 2000–2018; curated as in Temby et al. (2025) [dataset]

Journal Title	Papers (n)	Percent (%)
1. Proceedings of the Annual Offshore TechnologyConference	123	2.9
2. Proceedings - S.P.E. Annual Technical Conference and Exhibition	99	2.3
3. Marine Ecology Progress Series	84	2.0
4. PLoS One	79	1.9
5. Proceedings of the International Conference on Offshore Mechanics andArctic Engineering – OMAE	69	1.6
6. Estuaries and Coasts	57	1.3
7. Continental Shelf Research	53	1.2
8. Journal of Geophysical Research: Oceans	48	1.1
9. Environmental Science and Technology	46	1.1
10. (tie) Deep-Sea Research Part II: Topical Studies in Oceanography	45	1.1
11. (tie) Journal of Coastal Research	45	1.1
12. Bulletin of Marine Science	41	1.0
13. Geophysical Research Letters	39	0.9
14. (tie) Estuarine Coastal and Shelf Science	38	0.9
15. (tie) Marine Biology	38	0.9
16. (tie) Marine Pollution Bulletin	38	0.9

**Table 2:** Top 15 organizations publishing research on the Gulf of Mexico by the number of inter-organizationally co-authored papers (percent is out of 13,064 authors), followed by the number of organizations they collaborate with (2000–2018). *Links to other organizations* refers to the number of other organizations that authors of those organizations collaborated with. *Total links to other organizations* includes redundant links. Data source: Scopus (Elsevier), 2000–2018; curated as in Temby et al. (2025) [dataset]

Organization	Papers (*n*)	Authorship percent (%)	Links to other organizations	Total links to other organizations
1. National Oceanic and Atmospheric Administration (NOAA)	683	5.2	431	1325
2. Louisiana State University	333	2.6	263	751
3. Texas A&M University	307	2.4	320	738
4. US Geological Survey	305	2.3	257	613
5. University of Southern Mississippi	213	1.6	187	512
6. National Autonomous University of Mexico (UNAM)	211	1.6	206	373
7. University of South Florida	203	1.6	202	535
8. University of Texas at Austin	149	1.1	223	414
9. Florida State University	136	1.0	214	418
10. (tie) B.P. plc	133	1.0	129	233
10. (tie) University of Miami	133	1.0	158	390
12. University of South Alabama	131	1.0	128	281
13. Chevron Corp	128	1.0	110	200
14. Shell Corporation	125	1.0	112	182
15. University of North Carolina	120	0.9	195	414

### Inter-jurisdictional Collaboration

**Fig. 3:**
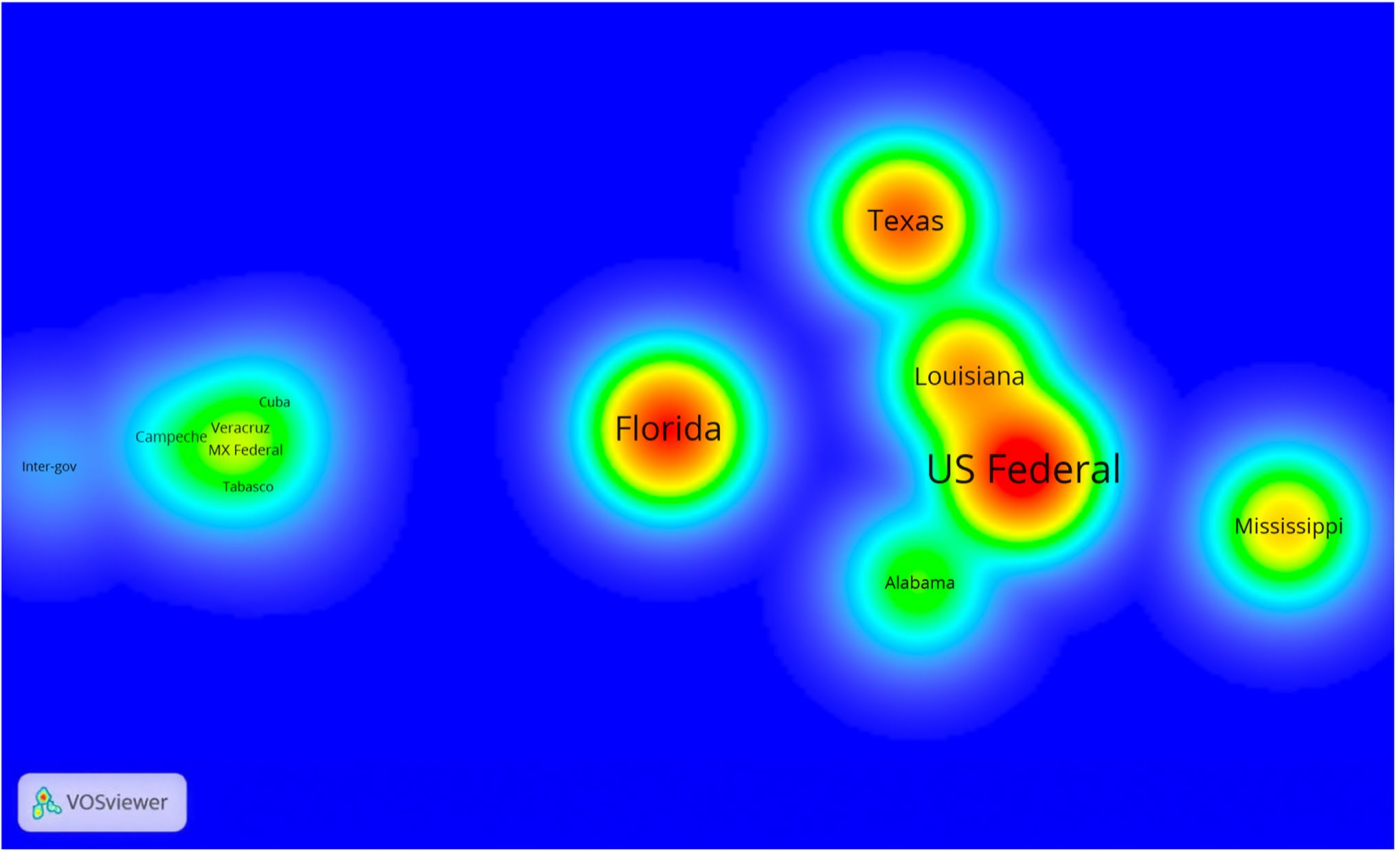
Inter-jurisdictional research collaboration among Mexican and U.S. coastal states and Cuban, Mexican, and U.S. federal governments in the Gulf of Mexico (2000–2018). Each node represents a political jurisdictional category. Node color indicates the level of collaboration (i.e., the redder the color the greater the number of co-authored papers produced by the organizations belonging to that jurisdiction). Distance between nodes denotes the intensity of collaboration (i.e., closely located nodes indicate a larger number of co-authored papers between them). Visualization tool: VOSviewer 1.6.0

A density diagram visualizing an inter-jurisdictional collaboration network is presented in Fig. 3. To highlight collaboration among regions geographically located on the Gulf, this diagram is restricted to Cuba, Mexico, and the U.S.A. and includes coastal Mexican and U.S. states only, plus national governments and inter-governmental organizations. The dark red node color of the U.S.A. indicates it has high levels of research collaboration. Its position in the center-right area of the mapped nodes highlights its potential role as a facilitator of science research occurring in the Gulf of Mexico region. Yet, it appears that U.S. collaboration occurs predominately between U.S.-located organizations and jurisdictions. Authors in the U.S.A. and Mexico collaborated on papers only 460 times, or 12% of all U.S. collaboration links. Additionally, the U.S.A. and Mexico only collaborated with Cuba 19 and 18 times, respectively. Cuba holds the lowest number of authorships (17) and one of the two lowest numbers of collaboration links (14). To add perspective: U.S.-Mexican co-authorship happened roughly as often as co-authorship between scholars in the U.S.A. and Canada (566 times), U.S.A. and UK (397 times), and the U.S.A. and Germany (269 times).

Considering Mexican and U.S. states, the results indicate high collaboration among U.S. states, with U.S. national-level authorship of 1784 papers. Texas and Florida hold intermediate to central locations in the map, reflecting their roles as facilitators of transboundary innovation and as producers of scientific knowledge. Of the total inter-organizational co-authorships, 1889 were located geographically in Texas among 304 organizations, and 1152 authorships were geographically located in Florida across 106 organizations. The Mexican states of Veracruz and Yucatan had the highest numbers of organizational co-authorships (126 and 119, respectively), while national-level organizations had 115. Excluded from this figure is Mexico City, a non-coastal entity that had more co-authorships (277) than the other Mexican states by virtue of it containing the internationally recognized National Autonomous University of Mexico. The distance between the Mexican jurisdictional category cluster and the cluster of U.S. states further depicts the weakness of these two countries’ collaboration. The strength of collaboration between inter-governmental organizations and both Mexican states and Cuba shows collaborative robustness that is stronger than either of the collaborative links that Mexican states or Cuba have with the U.S.A. Cuba has stronger ties with the Mexican states, with 29 total links.

### Inter-institutional Collaboration

**Fig. 4:**
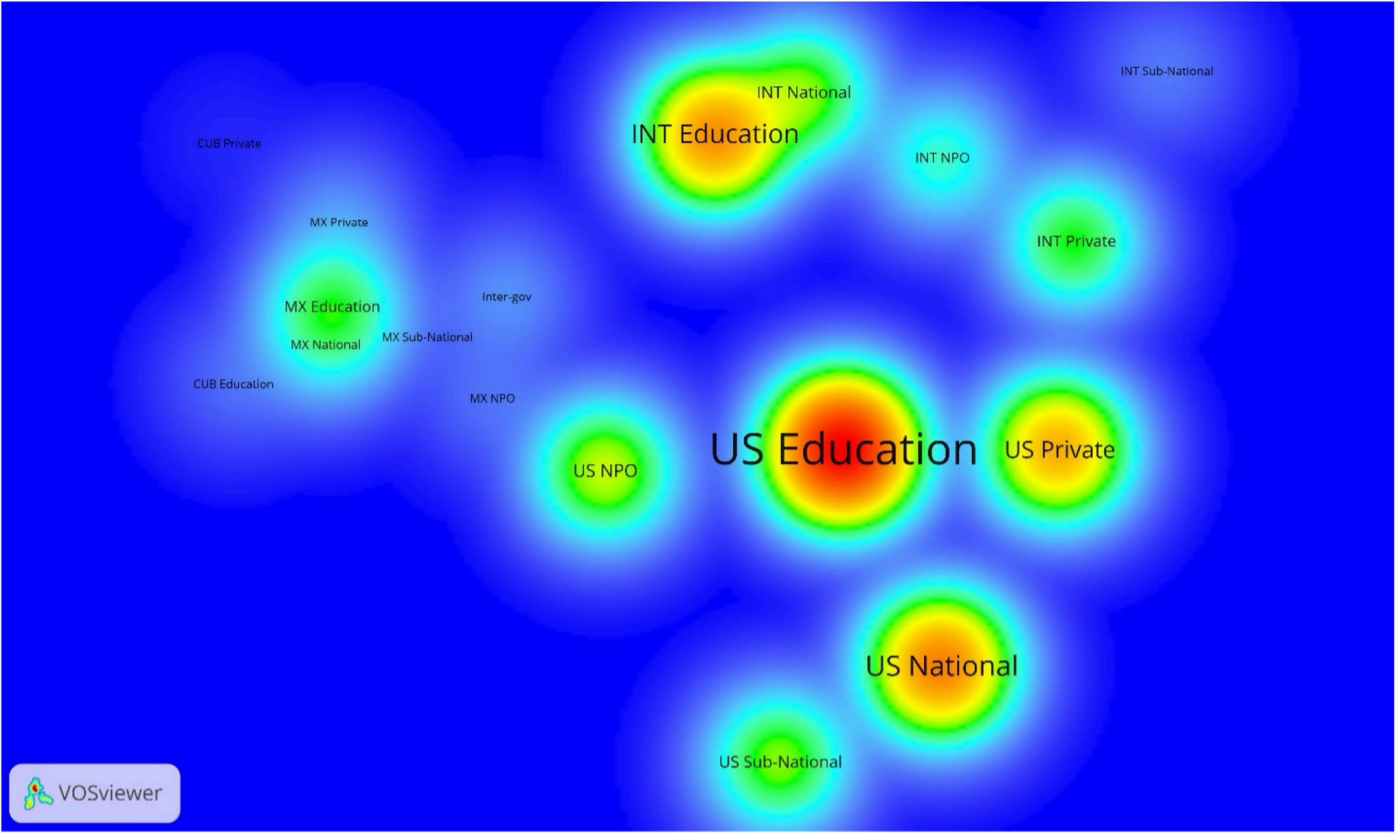
Inter-institutional research collaboration on the Gulf of Mexico (2000–2018). Each node represents an institutional category. Node color indicates the level of collaboration (i.e., the redder the color, the greater the number of co-authored papers produced by the organizations belonging to that institutional category). Distance between nodes denotes the intensity of collaboration (i.e., closely located nodes indicate a larger number of co-authored papers between them). Visualization tool: VOSviewer 1.6.0

Figure 4 shows the patterns of co-authorship among organizations grouped by institution type (e.g., educational institution, private firm) and nationality. The nationalities are the three countries of the Gulf of Mexico, plus “INT,” or international, representing all locations outside the three Gulf of Mexico countries. Educational institutions in the U.S.A. are at the center of co-authorship, representing roughly 40% of the 13,064 authorships. U.S. government agencies (i.e., US National) and private firms were frequent collaborators (about 14% and 15% of the authorships, respectively). U.S. non-profits and subnational government agencies also collaborated frequently with authors based in the U.S.A. but co-authored fewer total papers, each amounting to about 3% of authorships.

Among the other important authors of co-authored papers were those from Mexican educational institutions (about 6% of authorships) and authors from educational institutions outside the Gulf (nearly 11% of authorships). As with the U.S.A., there is a notable clustering among institutions from the different categories in Mexico. The inter-institutional density map also shows a clear fragmentation in paper authorship between the U.S.A., Mexico, and countries outside the region.

## Discussion

The results of our bibliometric analysis show that, although scientific collaboration is increasing in the Gulf of Mexico from 62 papers in 2000 to 372 in 2018 (Fig. 2), collaboration remains internationally fragmented among institutions located in Mexico, the U.S.A., and Cuba. The number of inter-institutionally co-authored papers related to environmental issues in the Gulf of Mexico has increased over the period of the study. The majority of collaborations occur between federal, private, and educational institutions. The Gulf of Mexico region produces large amounts of published work overall, with bibliometric search results double those produced in another large North American transnational coastal region (Song et al., 2015).

Despite the large amount of published work in the Gulf of Mexico, our results show that co-authorship between the U.S.A. and Mexico was limited, with U.S.-based scholars collaborating at comparable levels with scholars in Western countries outside the Gulf of Mexico region, such as Canada and the UK. The limited collaboration between the U.S.A. and Mexico is striking due to the binational policy efforts over border resources that have occurred over the past five decades (Cruz & McLaughlin, 2008; Wilder et al., 2020) such as the La Paz Agreement and the North American Agreement on Environmental Cooperation (Coronado & Mumme, 2020; Correa-Cabrera & Konrad, 2020).

Studies that highlight the relevance of specific differences among countries impeding research collaboration, such as language (Hoekman et al., 2010), may point to potential explanations for our finding of minimal U.S.A.-Mexico co-authorship. Wagner et al. (2001) show that scientifically advanced countries like the U.S.A. are more likely to collaborate with other advanced countries, while scientifically developing countries (such as Mexico and Cuba) are more likely to collaborate with each other. Wagner et al. (2001) attributed difficulties in collaboration between advanced and developing countries to differences in scientific capacity. Developing countries tend to specialize in science based on a specific national need while advanced countries focus on a wider range of fields (Wagner et al., 2001). Further, technological and organizational proximity were identified as key factors for collaboration (Lane & Lubatkin, 1998). Technological proximity is the level of overlap of the tools, devices, and knowledge bases of two collaborating actors (Knoben & Oerlemans, 2006; Lane & Lubatkin, 1998). Organizational proximity is defined by Rallet and Torre (1999, 375) as “the set of routines – explicit or implicit – which allows individuals of a same organisation to be co-ordinated without having to define beforehand how they must do it.”

The study results reveal that Cuba has minimal scientific collaboration with the U.S.A., but fairly robust inter-jurisdictional and inter-institutional scientific collaboration with Mexico. This may be attributable to their shared language or their relative closeness in organizational and technological proximity. To improve collaboration on environmental science and research in the Gulf of Mexico, there is an opportunity for researchers in the U.S.A. to strengthen its link with Cuba through Mexico. Collaboration between the U.S.A. and Cuba has mainly improved through informal efforts by academics, NGOs, and private foundations (Dean, 2007). Political relationships between Cuba and the U.S.A. have been improving since 2014, with President Obama’s administration initiating a major policy shift toward engagement and the normalization of relations (U.S. Congressional Research Service, 2021). This policy shift led to numerous bilateral agreements between U.S. and Cuban officials, including the 2017 Maritime Boundary Agreement (U.S. Congressional Research Service, 2021). However, President Trump’s administration rolled back some of the policy changes, and divisions persist in the U.S. Congress over foreign policies and strategy toward Cuba (U.S. Congressional Research Service, 2021). A more in-depth analysis of Cuba’s production of scientific knowledge and current collaborative programs is needed. Overall, this study suggests a need to promote cross-border scientific relations, which would facilitate further opportunities to collaborate.

Other studies provide insight into barriers inhibiting international research collaboration at the Gulf of Mexico scale, and how they might be attenuated (Hoekman et al., 2010; Wagner et al., 2001). To connect various jurisdictions and institutions, institutional constraints such as differing funding requirements, regulations, and assessment criteria need to be considered and reduced (Hoekman et al., 2010). Wagner et al. (2001) found that most scientists began collaborations because of in-person meetings and continued the formal or informal collaborations online thereafter. Thus, the opportunity for face-to-face interaction through research site visits or international conferences appears to be crucial for reducing inter-jurisdictional fragmentation. This would be particularly important for the U.S.A. and Cuba, which have historically banned travel between countries and made interactions difficult.

Our results show that NOAA is the largest producer of published collaborative research in the Gulf of Mexico and suggests that the agency may be uniquely positioned to foster cross-border scientific research. At the same time, the institutional arrangements that have historically supported such collaboration appear increasingly contingent. Recent shifts in federal priorities—including cancelled research contracts and administrative restructuring under the U.S. Department of Government Efficiency—have introduced new uncertainties into the landscape of environmental science. The Trump administration’s fiscal year (FY) 2026 discretionary budget request proposes a reduction in federal funding of 73.86% for NOAA research including the cancelling of all funds for Cooperative Institutes and termination of several federal contracts with Universities (Office of Management & Budget, 2025). The long-term viability of international research partnerships may depend less on formal mechanisms than on the strength of informal scholarly relationships and decentralized initiatives. Strengthening these connections, particularly among organizations in the U.S.A, Mexico, and Cuba, may provide a more durable foundation for scientific cooperation, especially when formal diplomacy is politically constrained or inconsistently applied.

## Data Availability

The dataset supporting this study is available in ScholarWorks@UTRGV at https://scholarworks.utrgv.edu/datasets/1.
